# Longitudinal Changes of Serum Creatine Kinase and Acute Kidney Injury among Patients with Severe COVID-19

**DOI:** 10.1155/2022/8556793

**Published:** 2022-04-26

**Authors:** Juan M. Soto-Fajardo, Valeria J. Castillo-Avalos, Elisa Naomi Hernandez-Paredes, Airy Santillán-Cerón, Jorge E. Gaytan-Arocha, Olynka Vega-Vega, Norma Uribe, Ricardo Correa-Rotter, Juan C. Ramirez-Sandoval

**Affiliations:** ^1^Department of Nephrology and Mineral Metabolism, Instituto Nacional de Ciencias Médicas y Nutrición Salvador Zubirán, Mexico City, Mexico; ^2^Department of Pathology, Instituto Nacional de Ciencias Médicas y Nutrición Salvador Zubirán, Mexico City, Mexico

## Abstract

**Background:**

Acute kidney injury (AKI) is a common complication of COVID-19. Several etiologies have been identified, including pigment deposition likely associated with myopathic damage. Nevertheless, the relationship between longitudinal creatine-kinase trends and renal outcomes is uncertain.

**Aim:**

To correlate longitudinal changes in serum creatine-kinase levels with hospital-acquired AKI (beyond 48 h of hospital admission) in severe COVID-19 patients.

**Methods:**

This is a retrospective cohort study, and creatine-kinase levels were assessed over time in 1551 hospitalized patients with normal renal function at the time of hospital admission.

**Results:**

In subjects who developed hospital-acquired AKI (*n* = 126, 8.1%), the serum creatine-kinase concentration before AKI onset was not different when compared to patients without AKI (slope of log creatine-kinase/day = −0.09 [95% CI −0.17 to +0.19] vs. +0.03 [95% CI −0.1 to +0.1]). After AKI diagnosis, serum creatine-kinase levels showed a significantly ascendent slope (slope of log creatine-kinase/day after AKI diagnosis = +0.14; 95% CI + 0.05 to +0.3). The AKI evolution was the main factor associated with the creatine-kinase trend. Subjects with persistent AKI (*n* = 40, 32%) had rising creatine-kinase levels during hospitalization (slope of log creatine-kinase/day = +0.30 95% CI + 0.19 to +0.51). A rising creatine-kinase trend (*n* = 114, 8%) was associated with a 1.89-fold higher risk of in-hospital death (95% CI 1.14 to 3.16). Nevertheless, this association disappeared after adjusting AKI evolution and LDH baseline levels.

**Conclusion:**

In severe COVID-19 patients, a slight increase in creatine-kinase levels was observed after AKI occurrence but not before. Our results show that, at least for the appearance of hospital-acquired AKI, the CK rise does not meet the temporality criterion of causality regarding the occurrence of AKI. Rising creatine-kinase trends were associated with a higher risk of mortality, but this association was modified by AKI evolution and inflammation. There is a limited efficiency for AKI prognosis in the serial follow-up of CK levels in severe COVID-19 patients with normal renal function.

## 1. Introduction

A diversity of manifestations related to kidney involvement in coronavirus disease 2019 (COVID-19) have been recognized as a prominent characteristic of SARS-CoV-2 infection, and acute kidney injury (AKI) is one of the most prevalent and present in 28% of hospitalized patients [1]. Multiple pathophysiological pathways of kidney injury have been described and manifested as tubular and glomerular pathology secondary to systemic hemodynamic instability, regional inflammation, endothelial damage, renal microthrombi, or direct viral invasion of the kidneys [2].

An additional potential mechanism of COVID-19 related AKI could be the deposition of endogenous heme pigment-containing proteins within renal tissue [3], commonly designated as *pigment nephropathy*. The inflammatory response to SARS-CoV-2 infection may induce the breakdown and deposition of endogenous heme pigment-containing proteins (myoglobin and hemoglobin). In addition, other potential risk factors for rhabdomyolysis commonly present in COVID-19 patients include physiological factors (exertion of the respiratory muscles, hypoxia, acidosis, and electrolyte imbalances [4]), medications (steroids, propofol, neuromuscular agents, and hydroxychloroquine) [5], prolonged immobilization, and mechanical ventilation, especially for patients in the prone position or treated with a permissive hypercapnia strategy [6]. In the most extensive postmortem kidney biopsy series of 85 deceased patients with COVID-19, the presence of pigment cast occurred in 54% and was associated to a significantly lower probability of kidney function recovery [7]. In hospitalized COVID-19 patients, serum creatine-kinase (CK) levels were independently associated with AKI diagnosis at the time of admission [8]. Nevertheless, the evidence about this potential link is scant [9].

Serum CK is one of the classical biomarkers of muscle cell injury and has traditionally been considered the best predictor of AKI in critically ill patients with rhabdomyolysis [10]. However, absolute CK cut-off values have been inconsistent in predicting AKI [11]; only very high CK levels (>40,000 IU/L) pose a significant risk associated with severe AKI and a higher mortality risk [12]. The chronological sequence of CK elevation (*CK trend*) has been suggested as a better marker for rhabdomyolysis [13]. The classical description depicts a gradual CK increase during the first 12 h of muscle damage, which peaks within 3–5 days, and a return to baseline during the following 10 days [14]. However, there is no evidence of a relationship between longitudinal serum CK measurement and renal outcomes. Analysis of longitudinal data is required to determine whether serum CK levels change in association with the onset, progression, and increasing severity of COVID-19 associated AKI.

Therefore, the aim of this study was to correlate longitudinal changes in serum CK levels with AKI evolution in hospitalized patients with severe COVID-19.

## 2. Materials and Methods

This is a single-center, retrospective, observational study conducted at the Instituto Nacional de CienciasMédicas y Nutrición Salvador Zubirán, a third-level healthcare center in Mexico City, designated to treat COVID-19 patients exclusively. We included all hospitalized COVID-19 patients who required supplemental oxygen from March 17, 2020, to March 1, 2021. COVID-19 was confirmed in all by RT-qPCR test from nasopharyngeal swabs and/or computerized tomography. All patients had severe COVID-19 as defined by National Institute of Health criteria [15]. The study was approved by the local Human Research and Ethics Committees (reference no. NMM-3795-21-22-1).

Clinical data were collected from electronic health records. We excluded patients who had elevated CK at the time of hospital admission due to renal disease (AKI diagnosis at the time of admission, development of AKI <48 h after hospital admission or previous diagnosis of chronic kidney disease (CDK)), cardiac etiology (myocardial infarction within 48 h of admission), and/or cerebral vascular accident/stroke/status epilepticus. In patients where preadmission serum creatinine was missing, we calculated a serum creatinine baseline concentration based on an imputed eGFR of 75 mL/min/1.73 m^2^ by the CKD-EPI formula [16], as recommended by the Acute Dialysis Quality Initiative (ADQI) [17]. For readmitted patients, the analysis only included data from the first hospitalization, in order to avoid data duplication. Other excluded subjects were those who requested voluntary discharge or were transferred to another institution.

The main predictor of interest was the in-hospital serum CK changes. Serum CK assessments were performed frequently during hospitalization since our institutional protocol established a laboratory test panel for hospitalized COVID-19 patients, which includes serum CK. Serum CK values were not available every single day in all patients as blood draws were not always performed daily. Serum CK levels were measured using the Architect CK reagent on Architect i1000SR from Abbott Diagnostics (reference range 30–223 IU/L).

The outcomes of interest were the *onset* and *evolution* of hospital-acquired AKI. Hospital-acquired AKI was defined as any AKI onset documented beyond 48 h of hospital admission. As urine output was absent in many cases, this criterion was not considered for AKI diagnosis. AKI and AKI stages were dependent on serum creatinine levels according to KDIGO (Kidney Disease: Improving Global Outcomes) criteria (serum creatinine increase ≥0.3 mg/dL) [18]. For *AKI evolution* endpoints, subjects were classified as follows: *persistent AKI*, *rapid reversal AKI,* or *recovery AKI.* “Persistent AKI” was operationally defined as the continuance of AKI due to elevated serum creatinine beyond 48 h from AKI onset, “rapid reversal AKI” was defined as a return to baseline serum creatinine values within 48 h of AKI onset, and “recovery AKI” was defined as a reduction in the peak AKI stage within 48 h not fulfilling the definition of rapid reversal of AKI [19]. A secondary outcome of interest considered was in-hospital mortality risk at 90 days after follow-up.

Continuous variable distribution was assessed using the Kolmogorov–Smirnov test. Descriptive statistics were expressed as a median (interquartile range (IQR)) for continuous variables and as a number (%) for categorical variables. Serum CK changes during in-hospital follow-up were estimated from separate linear mixed-effects models, each adjusted by age, sex, and body mass index. An intercept and slope over time were included as correlated random effects varying by subject in each model. Follow-up serum CK data were censored either when participants were discharged, initiated dialysis treatment, and died or went beyond April 1, 2021. Serum CK levels were transformed into a common logarithmic scale to improve model fit. ANOVA or Kruskal–Wallis and Chi-squared tests were used to compare continuous and categoric variables among different in-hospital CK trajectories. We performed trajectory analyses using group-based trajectory models [20], a method that facilitates the detection of multiple subpopulations with recognizable longitudinal patterns of serum CK concentrations, as it may be more important to distinguish biomarker trends over time in individual patients rather than mean changes in a population. Three categories of CK levels were defined for 1551 subjects: stable, rising, and fluctuating. Kaplan–Meier curves were used to display the risk of in-hospital mortality among different in-hospital CK trajectories. The hazard ratios and 95% confidence intervals for mortality in the rising CK group were estimated using the Cox regression model. Adjustment for baseline covariates included age, male sex, body mass index (kg/m^2^), the occurrence of hospital-acquired AKI, persistent AKI evolution, and laboratory parameters at the time of admission (PaO_2_/FiO_2_, protein-C reactive, LDH, ferritin, and D-dimer). All statistical tests were performed using two-sided 5% significance levels. Analyses were performed using Stata 15.0 (Stata Corp, College Station, TX USA) and GraphPad Prism 6.0 (GraphPad Software, San Diego, CA, USA).

## 3. Results

### 3.1. Clinical Characteristics

We identified 2,908 hospitalized patients with COVID-19 and at least two or more serum CK measurements during hospitalization. Of these, a total of 1551 subjects with 4244 serum CK measurements fulfilled inclusion criteria. Patients underwent an average of 4.5 ± 1 CK measurements. We excluded 1357 cases for several reasons: 987 (73%) had an AKI diagnosis at the time of admission or developed AKI <48 h after admission, 194 (14%) had a previous diagnosis of chronic kidney disease, 97 (7%) were transferred to another institution, 18 (1%) were readmitted following discharge, 32 (3%) requested voluntary discharge, and 29 (2%) remained hospitalized beyond April 1, 2021. Myocardial infarction occurred in 13 subjects and stroke occurred in one subject, but all of these cases had concomitant AKI at the time of admission.

Hospital-acquired AKI occurred in 126 (8.1%) patients, of whom 69 (55%), 21 (17%), and 36 (29%) had stage 1, 2, and 3 AKI, respectively, and only 14 (11%) required renal replacement treatment. Clinical characteristics of subjects with hospital-acquired AKI and without AKI are presented in Table 1. As shown, subjects with hospital-acquired AKI included a higher proportion of males and had higher levels of serum inflammatory biomarkers including LDH, C-reactive protein, ferritin, D-dimer, and sensitivity Troponin I (Hs-cTnI). Compared to subjects without AKI, those with hospital-acquired AKI had higher rates of in-hospital mortality (52.4% *vs* 11.7%; *P* < 0.001).

### 3.2. Longitudinal Serum CK Concentrations before and after Hospital-Acquired AKI

**Figure 1:**
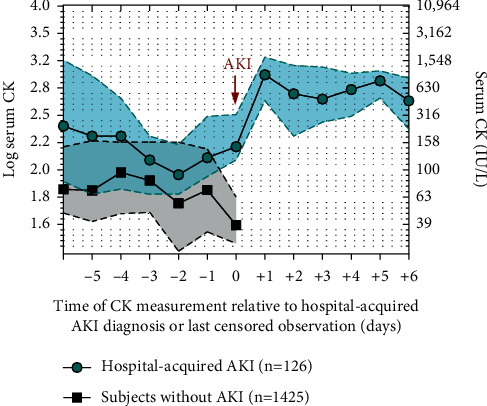
Longitudinal trajectory of serum CK measurements relative to the time for hospital-acquired AKI diagnosis or last censored observation. Green and black correspond to observed means of log serum CK concentrations (a) or CK concentrations in IU/L (b) for subjects with hospital-acquired AKI and without AKI respectively; 95% confidence intervals are represented in shaded areas.

Figure 1 shows longitudinal serum CK measurements relative to the time of hospital-acquired AKI diagnosis in those who developed this condition. In patients without AKI, longitudinal serum CK concentrations are depicted until the last censored measurement.

In subjects who developed hospital-acquired AKI, serum CK concentrations before AKI onset were not different when compared to patients without AKI diagnosis (slope of log serum CK per day in hospital-acquired AKI group before AKI diagnosis = −0.09; 95% CI −0.17 to +0.19 vs. slope of log serum CK per day in the nonAKI group = +0.03; 95% CI −0.1 to +0.1, *P*-value for group *∗* time interaction = 0.17). After a diagnosis of hospital-acquired AKI, serum CK levels showed a significantly ascendent slope (slope of log serum CK per day after AKI diagnosis = +0.14; 95% CI + 0.05 to +0.3). The slope of serum CK levels after AKI onset was significantly different when compared to serum CK slopes before AKI and to slopes of patients who did not develop AKI (*P*-value for group *∗* time interaction <0.001). Mean serum CK levels were 631 (95% CI 398 to 1584) IU/L after 48 hours from AKI diagnosis, compared to 151 (95% CI 125 to 316) IU/L measured on the day of AKI diagnosis (*P* < 0.001). CK levels >1000 IU/L in the hospital-acquired AKI group were observed for 17%, 48%, and 36% of patients after 24 h, 48 h, and 72 h of AKI onset, respectively. Among all hospitalized individuals, only 20 (0.5%) serum CK measurements were >5,000 IU/L and occurred more frequently in subjects with hospital-acquired AKI (*n* = 8 (6.3%) vs *n* = 12 (0.8%); *P* < 0.001).

### 3.3. Longitudinal Serum CK Concentrations and AKI Evolution

**Figure 2:**
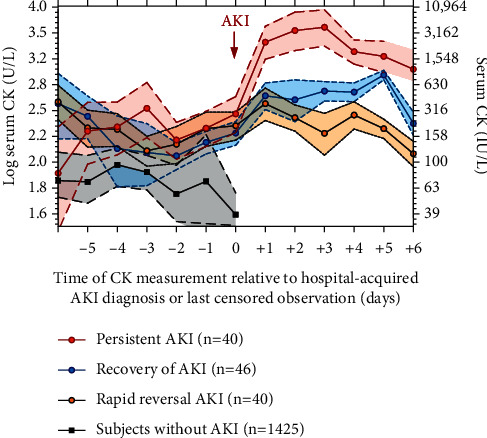
Longitudinal trajectory of serum CK measurements relative to the time of hospital-acquired AKI evolution or last censored observation. Red, blue, orange, and black represent means of log serum CK concentrations (a) or CK concentrations in IU/L (b) for subjects with *persistent AKI, rapid reversal AKI, and recovery AKI* and without AKI, respectively; 95% confidence intervals are represented in shaded areas.

After hospital-acquired AKI onset, AKI evolution was classified into three groups based on serum creatinine trajectory: *persistent AKI* (*n* = 40, 32%), *rapid reversal AKI* (*n* = 40, 32%), and *recovery AKI* (*n* = 46, 36%). As shown in Figure 2, patients with *rapid reversal of AKI* had a slight serum CK decrease after hospital-acquired AKI onset (slope of log CK per day = −0.08; 95% CI −0.02 to −0.15). In contrast, the mean and SD of serum CK rose more rapidly in patients with *persistent AKI*, indicating a gradual right skew in the distribution over time (slope of log CK per day = +0.30 95% CI + 0.19 to +0.51). The *recovery AKI* group had a relativity stable serum CK distribution over time (slope of log CK per day = +0.01; 95% CI −0.01 to +0.08). The serum CK slopes of each group according to AKI evolution were significantly different (*P* value for group *∗* time interaction <0.001).

### 3.4. Longitudinal Serum CK Concentrations and Mortality

**Figure 3:**
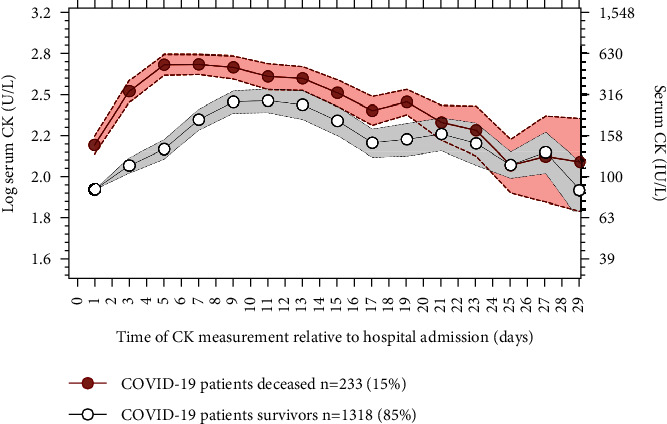
Longitudinal trajectory of serum CK measurements relative to hospital admission for COVID-19 patients, deceased *vs* survivors. Red (deceased) and black (survivors) correspond to means of log serum CK concentrations (a) or CK concentrations in IU/L (b) for COVID-19 deceased patients versus survivors; 95% confidence intervals are represented in shaded areas.

Across the cohort, in-hospital mortality was 15%. COVID-19 patients who died (233, 15%) had slope log serum CK concentrations, which were significantly higher than survivors (1318, 85%) during hospitalization (Figure 3).

### 3.5. Serum CK Trajectories and Outcomes

**Figure 4:**
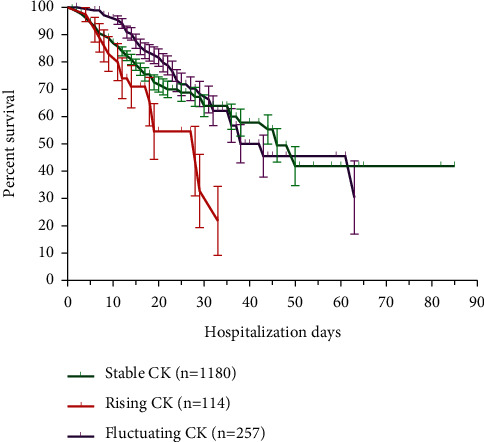
Kaplan–Meier curves for in-hospital mortality among COVID-19 subjects with rising CK, stable CK, and fluctuating CK trajectories.

Using group-based trajectory modeling, serum CK trajectories until mortality or discharge (whichever occurred first) were categorized into three main trajectories: (1) *stable* CK (*n* = 1180, 76%), (2) *rising* CK (*n* = 114, 8%), and (3) *fluctuating* CK (*n* = 257, 16%). Fluctuating CK included subpopulations with falling or bimodal (slowly rising and plateau) CK trajectories. Subjects with a *rising CK* trajectory had a higher proportion of obesity, hospital-acquired AKI, stage 3 AKI, renal replacement requirement, and in-hospital mortality. Likewise, this group had higher baseline levels of leucocytes, LDH, C-reactive protein, fibrinogen, D-dimer, and Hs-cTnI (Table 2). *Rising* CK trends were observed in 21/37 (57%) of hospital-acquired stage 3 AKI patients and in 25/40 (63%) patients categorized as *persistent AKI,* according to the *AKI evolution* classification. Survival curves for the main CK trajectories are shown in Figure 4. Patients with *rising CK* had a higher risk of mortality when compared to other CK trajectories (log rank *P* < 0.001). Patients with *stable CK* included a higher proportion of females and were associated with a lower inflammatory response and a higher survival probability.

**Figure 5:**
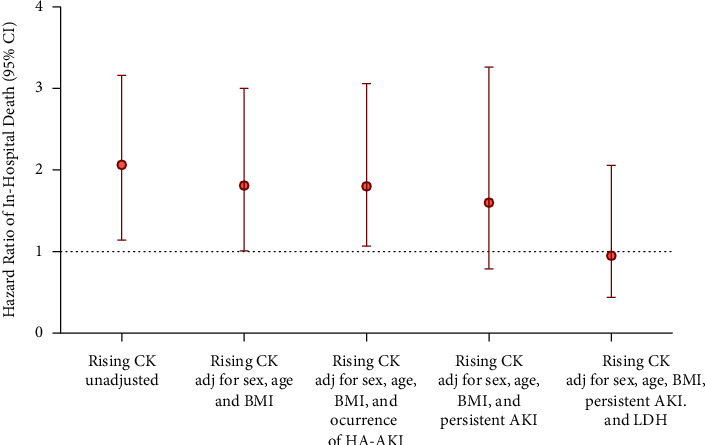
Unadjusted and adjusted hazard ratios for risk of death according to ascendent CK trajectory. Fully unadjusted and multivariable-adjusted hazard ratios and 95% confidence interval for mortality comparing the ascendent CK trajectory are shown. Adj, adjusted; BMI, body mass index; HA-AKI, hospital acute kidney injury; LDH, lactate dehydrogenase.

Proportional-hazard models were used to assess the risk of serum CK trajectories on the hazard ratio. Cox analyses for serum CK trajectories showed that belonging to a *rising* CK trajectory group was associated with a significantly increased risk of death (hazard ratio (HR) = 1.89, 95% CI = 1.14 to 3.16, *P* < 0.001). The HR for death was reduced from 1.9 to 1.6 (95% CI = 0.8 to 3.3) after adjusting the AKI evolution subcategory “persistent AKI”. This was further reduced to 0.9 (95% CI = 0.4 to 2.1) after adjusting levels of baseline serum LDH, an inflammation biomarker strongly associated with mortality in our cohort (Figure 5).

### 3.6. Other Outcomes

Postmortem kidney biopsy was performed in 6 of the cases included in the cohort, three of them with hospital-acquired AKI. None of the patients presented pigment casts in the biopsy, including 3 cases who were in the rising CK group (maximum CK level: 3,301 IU/L). In addition, the number of urine tests with “hemoglobin positive” and few red blood cells (suggestive of pigment nephropathy) within 48 h of AKI was less than 1% in all cohort (Table 2).

## 4. Discussion

In a large cohort of hospitalized patients with severe COVID-19, the analysis of longitudinal CK levels during hospitalization showed that (i) CK elevation was an event observed after AKI occurrence and not before, (ii) CK increased more frequently in severely ill COVID-19 patients with a high degree of inflammation, who had severe and persistent AKI, and (iii) although belonging to the rising CK group was associated with a higher mortality risk, this association was modified by other stronger risk factors, such as AKI evolution and the degree of inflammation assessed by LDH at the time of hospital admission.

In addition to traditional rhabdomyolysis risk factors, looking at CK trends has been suggested as a means to improve the prediction of AKI onset and progression [13]. The widespread use of CK levels is presently guiding therapeutic interventions in clinical practice to prevent pigment nephropathy, especially when CK elevations reach 5 times the upper limit of reference values [14]. However, there is controversy about the ability of CK threshold levels to predict renal failure. Our results indicate that CK concentrations are affected by glomerular filtration rate, especially in those with persistent AKI evolution. Although CK is eliminated by the liver and reticulum endothelial system, its kinetic is unknown in patients with decreased renal function [21]. De Meijer et al. previously described that kinetic analysis of CK levels correlates with the evolution of renal failure in patients with severe rhabdomyolysis [22]. The CK trajectory observed after AKI is very likely to occur in other critical illnesses (not COVID-19) with mild muscular and renal damage, but more studies using emerging analytic methods are needed.

There is uncertainty as to whether SARS-Cov2 infection or the current therapy for COVID-19 could increase the risk of myopathy [23]; therefore, the occurrence of pigment nephropathy contributes to kidney damage. In our cohort, more than three-quarters of subjects with severe COVID-19 had normal CK levels during hospitalization. In the remaining, most of the CK elevations occurred after the AKI event. In almost all hospital-acquired AKI cases, CK elevation was mild (<5000 IU/L). Assessing causalities is difficult in observational cohort studies, yet a great deal of emphasis has been placed on temporality. If the cause does not precede the effect, then the case for causality fails [24]. Our results show that, at least for the appearance of hospital-acquired AKI, the CK rise does not meet the temporality criterion regarding the occurrence of AKI. Likewise, the absence of *pigment nephropathy* in 6 cases of kidney biopsies, as well as the lack of hemoglobinuria without erythrocyturia in urine tests in follow-up, decreases the probability of *pigment nephropathy* presence.

Half of the COVID-19 patients with a rising CK pattern had a fatal outcome, yet only a fifth of them developed AKI, based on serum creatinine values before dying. Literature is limited regarding the inflammatory response in the musculoskeletal system caused by SARS-CoV-2 [25]. Although the rise of CK levels could be a specific prognostic marker, our interpretation of the data is that a CK increase is often a delayed finding, associated with AKI evolution and other inflammatory markers, limiting its usefulness in guiding the care for an individual patient. Similar results regarding CK values and mortality have been observed in other retrospective observational studies [11].

Strengths of this study include its large and well-characterized COVID-19 cohort with minimal follow-up loss, the number of measurements between exposure (CK trend) and the outcomes (serum creatinine levels), and the use of traditional and emerging analytic methods to evaluate repeated measurements.

The study has limitations. We use CK concentrations as a surrogate for pigment nephropathy, yet the use of myoglobin for this complication is recommended as a better biomarker [26]. The number of kidney biopsies was low in our cohort, although this is a common limitation in many AKI studies of critically ill patients. We cannot exclude that after AKI appearance, the rise of CK observed in patients with persistent AKI could be a magnifier of renal damage. Furthermore, we cannot discard subclinical tubular damage that might be present in patients with rising CK. The use of other specific tubular biomarkers in addition to serum creatinine could be helpful in future studies [27]. Likewise, the use of myoglobin and an increase in the number of kidney biopsies are needed to confirm our findings and conduct formal risk prediction analyses with new biomarkers.

## 5. Conclusion

Our results show that a moderate increase in CK levels occurs after a glomerular filtration decrease in severe COVID-19 patients, especially in seriously ill patients with a high degree of inflammation. Being in the rising CK group was a marker associated with mortality modified by AKI evolution and the degree of inflammation. Our analysis suggests that there is limited efficiency for AKI prognosis in the serial follow-up of CK levels in severe COVID-19 patients with normal renal function at the start of follow-up. The use of other new specific biomarkers validated for pigment nephropathy diagnosis in clinical practice is needed to improve a diagnosis and prevent AKI.

## Figures and Tables

**Table 1:** Cohort characteristics at the time of hospital admission.

Parameter at the time of hospital admission	Total *n* = 1551	HA-AKI *n* = 126 (8.1%)	NonAKI *n* = 1425 (91.9%)	*P* ^*∗*^
Age, years	52 (43–62)	53 (43–65)	52 (42–62)	0.33
Male sex, *n*(%)	949 (61)	88 (70)	861 (60)	0.04
Time since symptom onset, days	8 (6–11)	7 (5–10)	8 (6–11)	<0.001
DM, *n*(%)	380 (24.)	36 (29)	344 (24)	0.27
HTN, *n*(%)	366 (23)	36 (29)	330 (23)	0.17
Obesity, *n*(%)	633 (41)	54 (43)	579 (41)	0.62
BMI, kg/m^2^	29.1 (26.0–32.4)	29.4 (25.9–34.7)	29.1 (26.0–32.3)	0.6
Charlson comorbidity index, points	1 (0–2)	1 (0–2)	1 (0–2)	0.78
NEWS2 score, points	7 (6–8)	8 (7–9)	7 (6–8)	0.67
Serum creatinine, mg/dl	0.79 (0.69–0.93)	0.83 (0.71–0.93)	0.79 (0.69–0.93)	0.24
CK, IU/L	79 (44–163)	92 (52–185)	78 (43–163)	0.54
CK 1000–5000 IU/L, *n*(%)	24 (1.5)	14 (11)	10 (0.7)	<0.001
CK >5000 IU/L, *n*(%)	1 (0)	1(0.7)	0	1.00
LDH, IU/L (IQR)	327 (258–432)	422 (314–521)	321 (253–423)	<0.001
C-reactive protein, mg/dl	13.7 (7.2–20.6)	18.9 (12.0–25.3)	13.2 (7.0–20.2)	<0.001
Ferritin, mg/dl	525 (267–933)	629 (338–989)	521 (260–930)	<0.001
Hs-cTnI, pg/mL	4.8 (3.2–8.2)	7.9 (4.8–23.1)	4.7 (3.1–7.6)	<0.001
Fibrinogen, mg/dl	660 (490–774)	698 (540–838)	655 (485–770)	<0.001
D-dimer, ng/dl	755 (480–1198)	1008 (547–2154)	742 (476–1169)	<0.001
Hemoglobin, g/dl	15.4 (14.2–16.4)	15.3 (14.0–16.3)	15.4 (14.3–16.4)	0.14
Leucocytes, cells/mcl	8.2 (6–11.7)	10.2 (7.5–14.0)	8.1 (6.0–11.5)	<0.001
Total lymphocytes, cells/mcl	786 (549–1085)	685 (535–969)	793 (551–1087)	<0.001
PaO2/FiO2	222 (138–280)	158 (106–244)	226 (144–281)	<0.001

^*∗*^ *P* value shows a comparison between HA-AKI versus nonAKI groups. Quantitative data are presented as median (interquartile range). BMI, body mass index; CK, creatine phosphokinase; HA-AKI, hospital-acquired acute kidney injury; HTN, hypertension; Hs-cTnI, high-sensitive cardiac troponin I; LDH, lactate dehydrogenase; DM, diabetes mellitus. Obesity was defined as a BMI ≥30 kg/m^2^.

**Table 2:** Clinical characteristics stratified by serum CK trajectories.

	Rising CK *n* = 114 (8%)	Stable CK *n* = 1180 (76%)	Fluctuating CK *n* = 257 (16%)	*P* ^*∗*^
Age, years	50 ± 12	53 ± 14	50 ± 13	0.21
Male sex, *n*(%)	88 (77)	660 (55)	201 (78)	<0.001
Time since symptoms onset, days	8 (5–11)	8 (6–11)	8 (6–10)	0.89
DM, *n*(%)	24 (21)	303 (26)	53 (21)	0.15
HTN, *n*(%)	29 (25)	284 (24)	53 (21)	0.45
Obesity, *n*(%)	50 (44)	461 (39)	122 (47)	0.04
BMI, kg/m^2^	29.8 (27.5–32.5)	28.7 (25.8–32.3)	29.5 (27.1–33.0)	0.007
Baseline PaO2/FiO2	135 (85–164)	231 (152–288)	166 (104–256)	<0.001
Baseline creatinine, mg/dL	0.8 ± 0.2	0.8 ± 0.2	0.8 ± 0.2	1.00
Baseline Hb, g/dL	16.2 (14.4–17.0)	15.3 (14.2–16.4)	15.6 (14.6–16.4)	0.021
Leucocytes, cells/mcl	11.8 (7.6–15.0)	8.0 (5.9–11.4)	9.5 (6.4–12.8)	<0.001
Baseline LDH, IU/L (IQR)	488 (380–648)	309 (245–398)	410 (306–538)	<0.001
Baseline C-reactive protein, mg/dl	19 (12–26)	13 (7–20)	15 (9–22)	<0.001
Baseline ferritin, mg/dl	800 (337–1268)	489 (245–847)	669 (333–1164)	<0.001
Baseline Hs-cTnI, pg/mL	7.9 (4.4–21.9)	4.5 (3.0–7.4)	5.7 (3.7–9.2)	<0.001
Baseline fibrinogen, mg/dl	731 (586–897)	652 (482–770)	668 (524–802)	0.004
Baseline D-dimer, ng/dl	966 (523–1825)	733 (474–1150)	781 (517–1620)	<0.001
HA-AKI, *n*(%)	24 (21)	84 (7)	18 (7)	<0.001
**HA-AKI stage**				
Stage 3, *n*(%)	21 (18)	14 (2)	2 (1)	<0.001
Hemodialysis requirement, *n*(%)	7 (6)	7 (1)	0 (0)	<0.001
Urine with hemoglobin + &<5 RBC (AKI onset)	2 (2)	6 (0.5)	3 (1)	0.98
Urine with hemoglobin + &<5 RBC (48 h after AKI)	1 (0.8)	5 (0.4)	0 (0)	0.98
**HA-AKI evolution**				
Persistent AKI	10 (9)	27 (2)	3 (1)	<0.001
Rapid reversal AKI	2 (2)	29 (3)	9 (4)	0.54
Recovery AKI	12 (11)	28 (2)	6 (2)	<0,001
≥1 CK measurements ≥5000 IU/L, *n*(%)	20 (18)	0	0	<0.001
Mechanical ventilation, *n*(%)	110 (96)	210 (18)	109 (42)	<0.001
Death, *n* (%)	59 (52)	114 (10)	60 (23)	<0.001

^*∗*^ *P* value shows a comparison between groups. Quantitative data are presented as median (interquartile range). BMI, body mass index; CK, creatine phosphokinase; HA-AKI, hospital-acquired acute kidney injury; HTN, hypertension; Hs-cTnI, high-sensitive cardiac troponin I; LDH, lactate dehydrogenase; DM, diabetes mellitus; RBC, red blood cell. Obesity is defined as having a BMI of ≥30 kg/m^2^.

## Data Availability

All data generated or analyzed during this study are included in this article. Further enquiries can be directed to the corresponding author.
